# Randomized double-blind trial of beta-carotene and vitamin C in women with minor cervical abnormalities

**DOI:** 10.1038/sj.bjc.6690231

**Published:** 1999-03

**Authors:** D Mackerras, L Irwig, J M Simpson, E Weisberg, M Cardona, F Webster, L Walton, D Ghersi

**Affiliations:** Department of Public Health and Community Medicine, University of Sydney, Sydney, NSW, 2006, Australia; Menzies School of Health Research, Menzies School of Health Research, Darwin, NT, 0811, Australia; Family Planning NSW, Family Planning NSW, Ashfield, NSW, 2131, Australia

**Keywords:** cervical dysplasia, neoplasm, beta-carotene, vitamin C, randomized controlled trial

## Abstract

A double-blind, placebo-controlled, randomized, factorial study using a daily oral administration of 30 mg beta-carotene and/or 500 mg vitamin C was conducted in 141 women with colposcopically and histologically confirmed minor squamous atypia or cervical intra-epithelial neoplasia (CIN) I. Over approximately 2 years of follow-up, 43 lesions regressed to normal and 13 progressed to CIN II. The regression rate was slightly higher, but not significantly so, in those randomized to beta-carotene compared to no beta-carotene (hazard ratio = 1.58, 95% CI: 0.86–2.93, *P* = 0.14) and slightly lower, but not statistically significant, for those randomized to vitamin C compared to no vitamin C (hazard ratio = 0.65, 95% CI: 0.35–1.21, *P* = 0.17). In a model with no interaction, the progression rate was slightly higher in those randomized to beta-carotene (hazard ratio = 1.75, 95% CI: 0.57–5.36, *P* = 0.32) and also in those randomized to vitamin C (hazard ratio = 2.40, 95% CI: 0.74–7.80, *P* = 0.13). Neither of these were statistically significant. However, there was some evidence of an interaction effect of the two compounds on the progression rate (*P* = 0.052), with seven of the progressed lesions occurring in those randomized to both vitamins compared to a total of six in the three other groups. The currently available evidence from this and other trials suggests that high doses of these compounds are unlikely to increase the regression or decrease the progression of minor atypia and CIN I. © 1999 Cancer Research Campaign


*British Journal of Cancer* (1999) **79**(9/10), 1448–1453
© 1999 Cancer Research Campaign
Article no. bjoc.1998.0231

### Randomized double-blind trial of beta-carotene and vitamin C in women with minor cervical abnormalities

**D Mackerras[1,2], L Irwig[1], JM Simpson[1], E Weisberg[3], M Cardona[1], F Webster[1], L Walton[1] and D Ghersi[1]**

[1]Department of Public Health and Community Medicine, University of Sydney, NSW 2006, Australia; [2]Menzies School of Health Research, Darwin, NT 0811, Australia; [3]Family Planning NSW, Ashfield, NSW 2131, Australia

**Summary** A double-blind, placebo-controlled, randomized, factorial study using a daily oral administration of 30 mg beta-carotene and/or 500 mg vitamin C was conducted in 141 women with colposcopically and histologically confirmed minor squamous atypia or cervical intra-epithelial neoplasia (CIN) I. Over approximately 2 years of follow-up, 43 lesions regressed to normal and 13 progressed to CIN II. The regression rate was slightly higher, but not significantly so, in those randomized to beta-carotene compared to no beta-carotene (hazard ratio = 1.58, 95% CI: 0.86–2.93, $P$ = 0.14) and slightly lower, but not statistically significant, for those randomized to vitamin C compared to no vitamin C (hazard ratio = 0.65, 95% CI: 0.35–1.21, $P$ = 0.17). In a model with no interaction, the progression rate was slightly higher in those randomized to beta-carotene (hazard ratio = 1.75, 95% CI: 0.57–5.36, $P$ = 0.32) and also in those randomized to vitamin C (hazard ratio = 2.40, 95% CI: 0.74–7.80, $P$ = 0.13). Neither of these were statistically significant. However, there was some evidence of an interaction effect of the two compounds on the progression rate ($P$ = 0.052), with seven of the progressed lesions occurring in those randomized to both vitamins compared to a total of six in the three other groups. The currently available evidence from this and other trials suggests that high doses of these compounds are unlikely to increase the regression or decrease the progression of minor atypia and CIN I.

**Keywords:** cervical dysplasia; neoplasm; beta-carotene; vitamin C; randomized controlled trial

A variety of observational studies have found that dietary factors predict the incidence of cancer and precancerous lesions. Different sub-sets of these studies have been summarized to propose that beta-carotene or vitamin A is the active component (Ziegler, 1991; Hunter and Willett, 1994), that vitamin C is the active component (Block, 1991) or that the association is more consistent for fruit and vegetables as a group than for any particular nutrient (Steinmetz and Potter, 1991; Block et al, 1992). Some studies have used cervical intra-epithelial neoplasia (CIN) and invasive cervical cancer as the outcomes, but results disagree as to whether the strongest associations are for foods rich in beta-carotene and/or vitamin C and/or folate (Potischman, 1993). This inconsistency could be due to the differing accuracy of the dietary instruments for measuring the various vitamins, inadequate control for important confounders or because the nutrients are acting as markers for some other constituent in food. However, these studies do raise the possibility that increasing the intake of micronutrients might increase the regression rates or reduce the progression rates of minor cervical abnormalities – minor atypia and CIN I – to more severe abnormalities and cervical cancer. Although these nutrients are frequently described as antioxidants, some or all of them also exhibit other properties including cellular growth control, immunomodulation and gap junction modulation, which may be important after the initiation phase of carcinogenesis (Gerster, 1995). A variety of animal and in vitro studies have found that high doses of beta-carotene or vitamin C, given singly, have beneficial effects in retarding the promotion and progression of cancer (Gerster, 1995).

In 1991, we commenced a trial using 30 mg beta-carotene or 500 mg vitamin C daily in women with minor atypia or stage I CIN (CIN I) to investigate this possibility in humans. In this paper, we report the effect on the progression and regression rates during 2 years of follow-up.

### MATERIALS AND METHODS

We conducted a factorial, randomized, double-blind trial allocating equal numbers of women to an oral daily dose of either 30 mg beta-carotene or 500 mg vitamin C or both or neither. Subjects were stratified according to initial severity of the lesion (CIN I or minor squamous atypia) and randomized in permuted blocks of size 8 within strata. A diagnosis of the presence of human papilloma virus (HPV) alone was not considered sufficiently severe for inclusion in this trial. Minor atypia is a minimal change, not sufficient to be categorized as CIN I; it excludes inflammatory changes. Probable and definite CIN II are both referred to as CIN II hereafter.

The study was conducted at the Colposcopy Clinic in Sydney, Australia, run by Family Planning New South Wales (FPNSW) where clients with abnormal pap smears from six of the FPNSW clinics in Sydney are examined. All cytology slides are assessed by one cytology laboratory. The population using FPNSW services are better educated, more likely to be single and have higher status

*Received 6 March 1998*
*Revised 17 September 1998*
*Accepted 14 October 1998*

*Correspondence to:* D Mackerras, Menzies School of Health Research, Building 58, Royal Darwin Hospital, Rocklands Drive, Tiwi, NT 0811, Australia jobs than the general population (Young and Williamson, 1986). At the Clinic, a colposcopic examination and biopsy is performed by trained female medical staff who are judged competent by national audit criteria. All biopsies are assessed by one laboratory. At the start of the trial in 1991, the usual clinic policy was to advise women with CIN I that the 2-year progression rate was about 30% and that they could choose immediate ablation or 6-monthly follow-up by pap smear and colposcopy, with biopsy when clinically indicated. The majority of women in this clinic were choosing follow-up. Women with minor atypia were not offered the option of ablation and all were followed up at 6-monthly intervals by pap smear and colposcopy. Ethical approval for the trial was given by the University of Sydney Human Ethics Committee and the Ethics Committee of FPNSW on the understanding that women would make this choice before being informed of the existence of the trial. Sample size calculations were based on the accepted view at that time that a third of lesions would progress, a third would regress and a third would have no change over the 2-year duration (Campion et al, 1986). We estimate that 70 people would be needed in each of two arms comparing beta-carotene vs placebo or vitamin C vs placebo, in order to have 80% power to detect an increase in regression rate from 30% to 55%, and a reduction in progression rate from 30% to 10%, over 2 years as statistically significant at the 2-sided 5% level (Fleiss, 1981). As it was assumed the effects would be independent, a factorial design was used which gave a final sample size of 35 in each of four groups. The effect size of 2- to 3-fold was based on the results of the earlier observational studies (Block, 1991; Ziegler, 1991; Hunter and Willett, 1994).

Eligible women were those electing follow-up, aged 18 years or older, in whom no diagnosis of or treatment for a cervical abnormality had occurred in the previous 12 months. The diagnosis had to be based on having at least three of five assessments (one colposcopy and two independent assessments of each of the screening pap smear and the biopsy slides) grading the lesion as at least CIN I or minor atypia. Women were ineligible if any assessment was CIN III, if two assessments were CIN II or more severe, or if either reading of the histology slide returned a diagnosis of greater than CIN I; any women with abnormalities falling in these categories were contacted and offered treatment. These eligibility criteria were drawn up in recognition of the difficulties of assessing low-grade abnormalities of the cervix (Morrison et al, 1988) to ensure that there was a confirmed lesion at the start of the trial and to exclude those who might have CIN II. Additional eligibility criteria only ascertainable on interview were: anticipated residence in Australia for most of the next 2 years; no other health problems; not allergic to soya beans; no immediate plans for pregnancy; not taking more than 6 mg/day beta-carotene, 5000 IU/day vitamin A or 75 mg/day vitamin C routinely (high doses during a cold were acceptable). Routine use of other vitamins, minerals or herbal preparations was acceptable. If informed consent was obtained, a 1-month supply of capsules was left, with the explanation that it was a run-in period (but women were not told that these were placebo capsules). One month later, compliance and continued interest was assessed by phone call. If compliance was greater than 80%, the woman was randomized and sent a 6-month supply of study capsules; if less, a further run-in period was done if the woman was still interested.

A questionnaire was administered at the commencement of the run-in period ascertaining smoking levels and fruit and vegetable intake, using the summary questions from the Health Habits and History Questionnaire (Block, 1989), self-reported height, weight and smoking habit. Subjects were phoned every month to encourage and assess compliance and to ask about the incidence of various potential side-effects. Menstruation was ascertained as it had been decided that pregnant women, or women planning pregnancy, should be told to stop taking the capsules.

Subjects were seen in person by the research assistant at their 6-monthly visits to the Colposcopy Clinic. Compliance was assessed by pill-count at this visit. Clinic doctors knew the study was being conducted, but did not know which clients were in this study, as participants were not given any priority in the appointment system. After guidelines for treatment of patients were altered in 1994 (National Health and Medical Research Council, 1994), study participants' records had to be marked to allow continued management according to the former, stricter system for the duration of their participation in the trial. However, colposcopists remained blind to which group women were in. Subjects were free to withdraw from the study at any time. In accordance with Clinic policy, they could also elect to have ablation during the follow-up, despite no evidence of progression. Participants who decided to stop taking study capsules, but did not have ablation, were asked for permission to continue monitoring medical records for endpoints. As the 2-year point for the trial generally fell between examinations, participants were asked to continue taking capsules until the next examination.

At the end of the trial, all essential data were re-entered by a single individual who was blind to group allocation; medical records were examined and any unclear endpoints referred for review to a medical practioner, who was also blind to group assignment. Progression was deemed to have occurred when a result of CIN II or worse was obtained on any of pap smear, colposcopy or biopsy. Regression was defined as a normal result, with or without HPV, on both colposcopy and pap smear, on two successive clinic visits. Time to an endpoint was measured from the date of randomization to the date of the last clinic visit. The other categories of follow-up were: censoring at the final visit due to no change in the lesion, loss to follow-up, discharge following only one normal reading, ablation without evidence of progression, and withdrawal from the trial. Participants who ceased taking study capsules and agreed to permit us to monitor their records were followed until their 2-year visit; if permission was not given, the last visit to the colposcopy clinic prior to withdrawal was used as the withdrawal date.

Randomization was managed by the National Health and Medical Research Council Clinical Trials Centre, independently of the investigators. All capsules were identical in appearance and blister-packed in trays of 14 and marked with the names of the days to aid compliance. The randomization code for groups was not broken until after all statistical analyses had been completed. Synthetic vitamins in a soya bean oil base were manufactured with a 10% higher potency to allow for loss over time. Samples from the two storage locations were retested by the manufacturer (RP Scherer, Melbourne, Victoria) half-way through the trial in December, 1993 and contained, on average, 516.8 mg vitamin C and/or 30.8 mg beta-carotene.

An intention-to-treat analysis was done, using Cox's proportional hazards regression in BMDP (BMDP Statistical Software Inc, Los Angeles, CA) to test for interaction effects between the vitamins using maximum likelihood chi-square ($\chi^2$) statistics, and to estimate the hazard ratios and their 95% confidence intervals. The proportional hazards assumption was checked using log–log

© Cancer Research Campaign 1999

plots and by testing for an interaction between each variable and the logarithm of time to an endpoint. The assumptions held overall, although there was a suggestion that the assumption did not hold for progressions because the pattern of progressions in the groups differed in the first and second years of follow-up, but the small number of progressions made it difficult to assess. Progression and regression are competing risks, that is, women who experience one of these endpoints will not experience the other endpoint for a particular abnormality. Kaplan–Meier curves are the standard method for analysing time to an endpoint. However, this method incorrectly treats a woman who experiences a progression, for example, as censored when estimating the probability of a regression, and hence overestimates probability (Gaynor et al, 1993). Therefore, the probability curves for regression and progression were separately calculated from the cumulative incidence curves instead (Pepe and Mori, 1993).

### RESULTS

Of the 420 women invited to participate, 153 entered the run-in phase and 147 were randomized. Six did not return for follow-up, leaving 141 with analysable results. The first woman was randomized in August 1991 and the last in December 1993. The final follow-up visit occurred in November 1995.

Baseline characteristics, except the proportion of current smokers, were evenly distributed among the groups (see Table 1). There were 49 and 92 randomized in the CIN I and minor atypia strata respectively. There were 13 progressions to CIN II and 43 regressions to normal (Table 2), of which 10 and 29 respectively occurred before 2 years of follow-up. Among the remaining 102 participants, the median follow-up was 23 months: 50 completed 2 years or longer of follow-up, seven came up to 4 months early for their ‘2-year’ visit, 21 withdrew or were lost to follow-up, 13 were prematurely discharged from the Colposcopy Clinic, and 11 chose ablation despite no evidence of progression. All categories of censoring and drop-out were evenly distributed among the four groups. Of the 18 women who decided to stop taking study capsules, eight were in the double control group and the other 10 were evenly distributed among the other three groups. Overall, average compliance was 91%, not counting the period after women chose to discontinue study capsules, but including the interval while they were advised not to take them owing to planned or actual pregnancy.

**Table 1** Baseline characteristics of 141 randomized women by treatment groups (Sydney, 1991–93), showing mean (SD) or percentages

| | Control ($n$ = 35) | | Beta-carotene ($n$ = 36) | | Vitamin C ($n$ = 35) | | Both ($n$ = 35) | |
|---|---|---|---|---|---|---|---|---|
| Age (yrs) | 28 | (8) | 31 | (9) | 31 | (10) | 29 | (9) |
| Height (cm) | 164 | (7) | 166 | (6) | 165 | (7) | 166 | (7) |
| Weight (kg) | 60 | (9) | 62 | (9) | 61 | (9) | 61 | (12) |
| BMI (kg $m^{-2}$) | 22.2 | (3.3) | 22.5 | (3.2) | 22.5 | (4.0) | 22.2 | (3.6) |
| Fruit servings per week* | 11 | (7) | 9 | (6) | 13 | (7) | 10 | (5) |
| Vegetable servings per week** | 13 | (8) | 12 | (8) | 16 | (8) | 15 | (8) |
| Current smokers | 17% | | 25% | | 34% | | 37% | |
| Taking other vitamins & minerals | 37% | | 31% | | 31% | | 37% | |

*Servings of fruit excluding juice; **servings of vegetables excluding potatoes and salad.

**Table 2** Number of person-years of follow-up, regressions and progressions, and hazard ratios for regression and progression with and without interaction, by randomized group (Sydney, 1993–5)

| | | Person-years at risk | | | Number of regression events | | | Regression hazard ratio (95% CI) | | | Number of progession events | | | Progression hazard ratio (95% CI) | | |
|---|---|---|---|---|---|---|---|---|---|---|---|---|---|---|---|---|
| Vitamin C | | No | Yes | Total | No | Yes | Total | No | Yes | Total | No | Yes | Total | No | Yes | Total |
| Beta-carotene | No | 59 | 61 | 121 | 10 | 9 | 19 | 1 | 0.77 | 1 | 3 | 2 | 5 | 1.00 | 0.64 | 1.00 |
| | | | | | | | | referent | (0.21, 2.58) | referent | | | | referent | (0.11, 3.84) | referent |
| | Yes | 56 | 51 | 108 | 16 | 8 | 24 | 1.79 | 1.02 | 1.58 | 1 | 7 | 8 | 0.32 | 2.57 | 1.75 |
| | | | | | | | | (0.80, 4.01) | (0.40, 2.60) | (0.86, 2.93) | | | | (0.03, 3.12) | (0.66, 9.98) | (0.57, 5.36) |
| Total | | 116 | 113 | 229 | 26 | 17 | 43 | 1 | 0.65 | | 4 | 9 | 13 | 1.00 | 2.40 | |
| | | | | | | | | referent | (0.35, 1.21) | | | | | referent | (0.74, 7.80) | |

The person-years at risk table shows the total length of exposure to each treatment combination in person-years for the 2-year study. For the hazard ratio tables, the figures labelled ‘Total’ show the ratio of the hazard (e.g. of progression) in the treated group relative to the corresponding placebo group if no interaction is assumed (e.g. 1.75 for beta-carotene; 2.40 for vitamin C) while the internal cells show the hazard ratios relative to the double placebo group if an interaction is assumed (e.g. 2.57 for both treatments combined but 0.32 and 0.64 respectively for beta-carotene and vitamin C alone).

*© Cancer Research Campaign 1999*

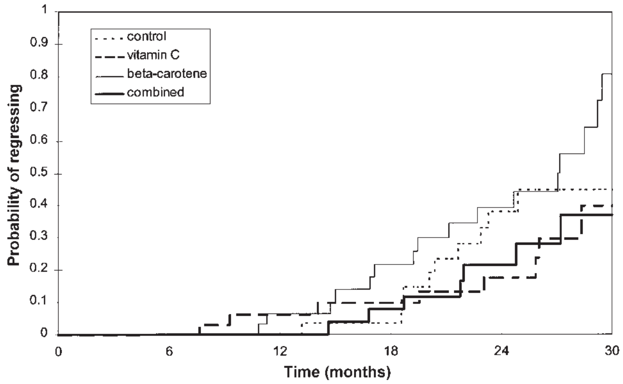

**Figure 1** Cause-specific curves showing the probability of a lesion regressing in each treatment group. The numbers of women in the study at entry and every 6 months thereafter were: 141, 132, 106, 88, 48, 9

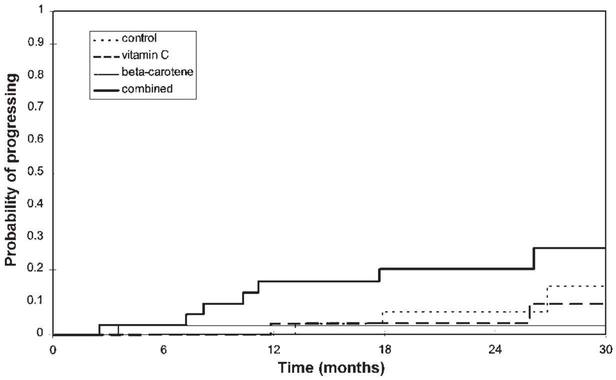

**Figure 2** Cause-specific curves showing the probability of a lesion progressing in each treatment group. The numbers of women in the study at entry and every 6 months thereafter were: 141, 132, 106, 88, 48, 9

The number of regressions and progressions by intervention group is shown in Table 2. The most notable observation is that seven of the progressions were in the groups receiving both vitamin C and beta-carotene, compared to a total of six progressions in the other three groups. This occurred even though the person-years at risk was slightly less in the group receiving both vitamins than in the other groups. To quantify the magnitude and statistical significance of this observation and other possible associations, hazard ratios (HR) and their 95% confidence intervals (CI) for both outcomes, with and without an interaction effect, are also shown in Table 2. Those randomized to beta-carotene had slightly higher, non-significant, regression rates (HR = 1.58, 95% CI: 0.86–2.93, $P$ = 0.14) than those not randomized to beta-carotene and those randomized to vitamin C had slightly lower, non-significant, regression rates (HR = 0.65, 95% CI: 0.35–1.21, $P$ = 0.17) than those not randomized to vitamin C (Table 2 and Figure 1). There was no evidence of an interaction between the compounds on the regression rate ($P$ = 0.64). In a model with no interaction, there was a non-significant slightly higher progression rate in those randomized to beta-carotene (HR = 1.75, 95% CI: 0.57–5.36, $P$ = 0.32) or vitamin C (HR = 2.40, 95% CI: 0.74–7.80, $P$ = 0.13). There was a statistically significant interaction effect ($P$ = 0.052), indicating that those receiving a combined dose of both beta-carotene and vitamin C were more likely to progress than those receiving neither (HR = 2.57, 95% CI: 0.66–9.98; Table 2 and Figure 2). Although not statistically significant, the 95% confidence intervals rule out large beneficial effects of either compound on the progression rate. None of these results was altered by adjusting for differences in baseline smoking prevalence.

### DISCUSSION

Since the commencement of our trial, several randomized controlled trials have reported on the effect of beta-carotene on various grades of cervical lesions. Using a 10 mg day$^{-1}$ dose in a randomized trial with a 3-month follow up, de Vet et al (1991) found a small, non-significant difference in regression that changed direction depending on whether the broad or strict definitions of regression were used (see Table 3). Using a longer follow-up in women with less severe lesions, neither Fairley et al (1996) nor Romney et al (1997) found a difference in regression rates over 9–12 months with a daily dose of 30 mg. Fairley et al (1996) found no difference in the quantity of DNA from human papilloma virus between the groups and Romney et al (1997) found a non-significant adverse effect of beta-carotene on persistence of HPV. Based on the four randomized trials, the results of Manetta et al (1996), who found a 70% regression of CIN I and II over 6 months in an uncontrolled trial of beta-carotene, must be regarded as being due to spontaneous regression rather than a positive effect of beta-carotene.

None of the studies, including ours, had many progression endpoints even in the control groups. However, the slightly favourable, non-significant, effect of beta-carotene on regression rates in our study needs to be viewed together with the slightly unfavourable, non-significant, effect on progression rates.

There are several important differences among the four studies. Firstly, we excluded anyone who had a histological diagnosis of CIN II or III whereas about two-thirds of the Dutch subjects (de Vet et al, 1991) and half the American subject (Romney et al, 1997) had these more severe lesions. Our study subjects generally had more severe lesions than those of Fairley et al (1996) and we did not allow the presence of HPV alone as a sufficient entry criterion. In addition, our entry criteria were based on three forms of assessment. We also had a considerably longer follow-up and a more stringent criterion for assessing regression than the other studies. We required a normal cervix to be documented on two successive pap smear and colposcopic examinations, whereas the other trials required only one examination, and some included regression to a less severe lesion as a regression endpoint. Whatever the differences in design, the studies collectively suggest that there is unlikely to be a protective effect of beta-carotene on the natural history of cervical precancers.

The pilot study for this trial showed an increase in beta-carotene levels from 2.7 (SD 2.3) μmol l$^{-1}$ to 4.1 (SD 2.3) μol l$^{-1}$ over 2 months. As expected, retinol levels did not change substantially (2.5 ± 0.6 μmol l$^{-1}$ at baseline; 2.6 ± 0.5 μmol l$^{-1}$ at 2 months). Others have reported that beta-carotene levels in serum and cervical cells increase to a maximum after 3 months when a 30 mg dose is used (Manetta et al, 1996). Hence, lack of an effect should not be attributed to lack of transport of beta-carotene into the cervix.

The non-significant effects of vitamin C in decreasing regression rates and increasing progression rates are both in

© Cancer Research Campaign 1999

**Table 3** Previous trials of beta-carotene and CIN

| | de Vet et al (1991) | | Fairley et al (1996) | | Romney et al (1997) | |
|---|---|---|---|---|---|---|
| | β-carotene 30 mg/day (synthetic) | Placebo | β-carotene 30 mg/day (from D salina) | Lecithin 400 mg day$^{-1}$ | β-carotene 30 mg/day (synthetic) | Lactose |
| Baseline lesion (*n*) | | | | | total group: | |
| Atypia | – | – | 1 | 4 | – | |
| HPV | – | – | 36 | 33 | – | |
| CIN I | 36 | 41 | 16 | 10 | 35 | |
| CIN II | 57 | 61 | 6 | 5 | 32 | |
| CIN III | 44 | 39 | – | – | 2 | |
| Follow-up | 3 months | | 12 months | | 9 months | |
| Change in cytology [*n* (%)] | | | | | | |
| Improved | – | – | 37 (63%) | 31 (60%) | 18 (46%) | 15 (50%) |
| No change | – | – | 19 (32%) | 17 (33%) | 20 (51%) | 14 (46.6) |
| Worse | – | – | 3 (5%) | 4 (7%) | 1 (2.6) | 1 (3.3) |
| OR (95% CI) for regression | | | | | | |
| Broad definition* | 0.68 (0.28–1.60) | | – | – | – | – |
| Strict definition* | 1.22 (0.43–3.41) | | – | – | – | – |

*The strict definition only included changes greater than one category; the broad definition included smaller changes.

unfavourable directions in our study. This seems to be the first trial to investigate the effect of vitamin C, either alone or in conjunction with beta-carotene, on cervical dysplasia, although a small quantity of 10 mg of vitamin C was used as the placebo in a trial investigating the effect of folic acid (Butterworth et al, 1982). One suggestion from our study is of a possible interaction between beta-carotene and vitamin C in increasing the progression rate. Vitamin C is found in the aqueous phase whereas beta-carotene is found on the inside cell membranes and tocopherols on the surface of cell membranes. Using a soybean lipoxygenase model, Niki et al (1995) have recently concluded that there should be no interaction between vitamin C and beta-carotene as antioxidants. However, our results support the possible existence of other mechanisms whereby an interaction might take place.

Beta-carotene has been tested in several other epithelial cancers. Various studies have found reductions in oral leukoplakia using beta-carotene, especially when combined with retinol, in subjects using tobacco or alcohol (Garewal et al, 1993). However lung cancer rates were slightly higher in Finnish smokers given beta-carotene (Alpha-tocopherol Beta-carotene Prevention Study Group, 1994) but overall cancer incidence and death rates were not raised in groups containing fewer smokers (Greenberg et al, 1996; Hennekens et al, 1996). In trials of people with a history of asbestos exposure, a combined dose of beta-carotene and retinol led to a slightly elevated incidence of lung cancer (Omenn et al, 1996) whereas another study found no differences in sputum atypia over a shorter period (McLarty et al, 1995). There does not seem to be another trial that has used beta-carotene combined with vitamin C. Trials using adenomatous colonic polyps as the outcome have tested various other combinations of beta-carotene, vitamin C, vitamin E and retinol (McKeown-Eyssen et al, 1988; DeCosse et al, 1989; Roncucci et al, 1993; Greenberg et al, 1994; MacLennan et al, 1995). Only Roncucci et al (1993), who used 1000 mg vitamin C plus 70 mg alphatocopherol plus 9 mg retinol (not beta-carotene), have found a favourable effect on the recurrence rate of adenomatous polyp.

The doses used in this and other studies were much higher than could be obtained from the diet. For example, Brown et al (1989) have shown that the rise in serum beta-carotene following a 12 mg dose of beta-carotene as a supplement is substantially higher than 29 mg consumed in carrots. This raises the possibility that the low intakes of beta-carotene and/or vitamin C described in epidemiological studies are risk markers rather than causal factors. It is also possible that intakes at the upper level of the dietary range throughout life may be beneficial, whereas pharmacological doses are inactive or deleterious. Our study does not address these questions. However, our results do contradict those of animal and in vitro work suggesting that high doses of these compounds would reduce the promotion of carcinogenesis.

The findings of our study and others provide a reminder of the importance of conducting randomized trials in humans rather than assuming that small, probably confounded, associations and animal models are sufficient information on which to base therapeutic or preventive recommendations. In view of the lack of effect, it is worth noting that the public is receiving different information from other sources. A number of women eligible for our trial had already decided to take antioxidant vitamins and did not wish to be randomized with the possibility of receiving the placebo. The currently available evidence suggests that high doses of these compounds are unlikely to increase the regression or decrease the progression of minor atypia and CIN I.

### ACKNOWLEDGEMENTS

We acknowledge the assistance of Jackie Brighton and Elaine Beller, NHMRC Clinical Trials Centre, for managing the randomization process; the staff of laboratories managed by Dr Colin Laverty and Dr Frank Pacey for re-reading histology and cytology

 *© Cancer Research Campaign 1999*

slides; the receptionists and doctors of the FPNSW Colposcopy Clinic for their contributions; the study participants, and comments from Drs Bruce Armstrong, Colin Laverty and Frank Bowden. This trial was funded in part by a grant from the National Health and Medical Research Council of Australia. Roche Products Pty Ltd supplied the vitamin preparations free of charge.

### REFERENCES

Alpha-tocopherol, Beta-carotene Prevention Study Group (1994) The effect of vitamin E and beta-carotene on the incidence of lung cancer and other cancers in male smokers. *New Engl J Med* **330**: 1029–1035

Block G (1989) *Health habits and history questionnaire*: *diet history and other risk factors*. *Personal computer system packet*. National Cancer Institute: Bethesda, MD.

Block G (1991) Vitamin C and cancer prevention: the epidemiologic evidence. *Am J Clin Nutr* **53**: 270S–282S

Block G, Patterson B and Subar A (1992) Fruit, vegetables, and cancer prevention: a review of the epidemiological evidence. *Nutr Cancer* **18**: 1–29

Brown ED, Micozzi MS, Craft NE, Bieri JG, Beecher G, Edwards BK, Rose A, Taylor PR and Smith JC (1989) Plasma carotenoids in normal men after a single ingestion of vegetables of purified β-carotene. *Am J Clin Nutr* **49**: 1258–1265

Butterworth CE, Hatch KD, Gore H, Mueller H and Krumdieck CL (1982) Improvement in cervical dysplasia associated with folic acid therapy in users of oral contraceptives. *Am J Clin Nutr* **35**: 73–82

Campion MJ, McCance DJ, Cuzick J and Singer A (1986) Progressive potential of mild cervical atypia: prospective cytological, colposcopic, and virological study. *Lancet* **ii**: 237–240

DeCosse JJ, Miller HH and Lasser ML (1989) Effect of wheat fiber and vitamins C and E on rectal polyps in patients with familial adenomatous polyposis. *J Natl Cancer Inst* **81**: 1290–1297

de Vet HCW, Knipschild PG, Willebrand D, Schouten HJA and Sturmans F (1991) The effect of beta-carotene on the regression and progression of cervical dysplasia: a clinical experiment. *J Clin Epidemiol* **44**: 273–283

Fairley CK, Tabrizi SN, Chen S, Baghurst P, Young H, Quinn M, Medley G, McNeil JJ and Garland SM (1996) A randomised clinical trial of beta-carotene vs placebo for the treatment of cervical HPV infection. *Int J Gynecol Cancer* **6**: 225–230

Fleiss J (1981) *Statistical methods for rates and proportions*, 2nd edn. John Wiley and Sons: New York

Garewal HS, Meyskens F Friedman S, Alberts D and Ramsey L (1993) Oral cancer prevention: the case for carotenoids and anti-oxidant nutrients. *Prev Med* **22**: 701–711

Gaynor JJ, Feuer EJ, Tan CC, Wu DH, Little CR, Straus DJ, Clarkson BD and Brennan MF (1993) On the use of cause-specific failure and conditional failure probabilities: examples from clinical oncology data. *J Am Statist Assoc* **88**: 400–409

Gerster H (1995) B-carotene, vitamin E and vitamin C in different stages of experimental carcinogenesis. *Eur J Clin Nutr* **49**: 155–168

Greenberg ER, Baron JA, Tosteson TD, Freeman DH, Beck GJ, Bond JH, Colacchio TA, Coller JA, Frankl HD, Haile RW, Mandel JS, Nierenberg DW, Rothstein R, Snover DC, Stevens MM, Summers RW and van Stolk RV for the Polyp Prevention Study Group (1994) A clinical trial of antioxidant vitamins to prevent colorectal adenoma. *New Engl J Med* **331**: 141–147

Greenberg ER, Baron JA, Karagas MR, Stukel TA, Nierenberg DW, Stevens MM, Mandel JS and Haile RW (1996) Mortality associated with low plasma concentration of beta carotene and the effect of oral supplementation. *JAMA* **275**: 699–703

Hennekens CH, Buring JE, Manson JE, Stampfer MJ, Rosner B, Cook NR, Belanger C, LaMotte F, Gaziano JM, Ridler PM, Willett WC and Peto R (1996) Lack of effect of long-term supplementation with beta carotene on the incidence of malignant neoplasms and cardiovascular disease. *New Engl J Med* **334**: 1145–1149

Hunter DJ and Willett WC (1994) Vitamin A and cancer: epidemiological evidence in humans. In *Vitamin A in health and disease*, Blomhof R. (ed) Marcel Dekker Inc: New York

McKeown-Eyssen G, Holloway C, Jazmaji V, Bright-See E, Dion P and Bruce WR (1988) A randomized trial of vitamins C and E in the prevention of recurrence of colorectal polyps. *Cancer Res* **48**: 4701–4705

McLarty JW, Holiday DB, Girard WM, Yanagihara RH, Kummet TD and Greenberg SD (1995) B-carotene, vitamin A and lung cancer chemoprevention: results of an intermediate endpoint study. *Am J Clin Nutr* **62**(suppl): 1431S–1438S

MacLennan R, Macrae F, Bain C, Battisuta D, Chapuis P, Gratten H, Lambert J, Newland RC, Ngu M, Russel A, Ward M, Wahlqvist ML and the Australian Polyp Prevention Project (1995) Randomized trial of intake of fat, fibre, and beta carotene to prevent colorectal adenomas: the Australian Polyp Prevention Project. *J Natl Cancer Inst* **87**: 1760–1766

Manetta A, Schubbert T, Chapman J, Schell MJ, Peng Y, Liao SY and Meyskens FJ (1996) Beta-carotene treatment of cervical intraepithelial neoplasia: a phase II study. *Cancer Epidemiol Biomarkers Prev* **5**: 929–932

Morrison BW, Erickson EW and Doshin Russo JF (1988) The significance of atypical cervical cytology. *J Reprod Med* **33**: 809–812

National Health and Medical Research Council (1994) *Screening to prevent cervical cancer*: *guidelines for the management of women with screen detected abnormalities*. Australian Government Publishing Service: Canberra.

Niki E, Noguchi N, Tsuchihashi H and Gotoh N (1995) Interaction among vitamin C, vitamin E and B-carotene. *Am J Clin Nutr* **62**(suppl): 1322S–1326S

Omenn GS, Goodman GE, Thornquist MD, Balmes J, Cullen MR, Glass A, Keogh JP, Meyskens FL, Valanis B, Williams JH, Barnhart S and Hammar S (1996) Effects of a combination of beta carotene and vitamin A on lung cancer and cardiovascular disease. *New Engl J Med* **334**: 1150–1155

Pepe MS and Mori M (1993) Kaplan-Meier, marginal or conditional probability curves in summarizing competing risks failure time data? *Stat Med* **12**: 737–751

Potischman N (1993) Nutritional epidemiology of cervical neoplasia. *J Nutr* **123** (2 suppl): 424–429

Romney SL, Ho GYF, Palan PR, Basu J, Kadish AS, Klein S, Mikhail M, Hagan RJ, Chang CJ and Burk RD (1997). Effects of B-carotene and other factors on outcome of cervical dysplasia and human papillomavirus infection. *Gynecol Oncol* **65**: 483–492

Roncucci L, DiDonato P, Carati L, Ferrari A, Perinin M, Bertoni G, Bedogni G, Paris B, Savoni F, Girola M and Ponz de Leon M (1993) Antioxidant vitamins or lactulose for the prevention of the recurrence of colorectal adenomas. *Dis Colon Rectum* **36**: 227–234

Steinmetz KA and Potter JD (1991) Vegetables, fruit, and cancer. I. Epidemiology. *Cancer Causes & Control* **2**: 325–357

Young L and Williamson A (1986) *Patterns of usage of Family Planning Association Clinics in the greater Sydney area*. Internal FPA report.

Ziegler RG (1991) Vegetables, fruits and carotenoids and the risk of cancer. *Am J Clin Nutr* **53**: 2518–2598

*© Cancer Research Campaign 1999*
